# Supplementary material for: A film-based intervention to reduce child maltreatment among migrant and displaced families from Myanmar: Protocol of a pragmatic cluster randomized controlled trial
Source: PLoS One. 2023 Oct 30;18(10):e0293623. doi: 10.1371/journal.pone.0293623 (PMC10615270; doi:10.1371/journal.pone.0293623)
Supplement: S1 File — (DOCX) [file pone.0293623.s001.docx]

S1 Fig. SPIRIT flow diagram: Schedule of enrolment, interventions, and assessments.

| **ACTIVITY** | **STUDY PERIOD** | | | | |
| --- | --- | --- | --- | --- | --- |
|  | **Enrolment** | **Baseline** | **Allocation** | **Post-allocation** | |
| **TIMEPOINT** | Jan-Jun 2023 | Feb-Jun 2023 | Apr-Jun 2023 | Jun-Sep 2023 | Aug-Dec 2023 |
| **ENROLMENT:** |  | | | | |
| Eligibility screen and recruitment of communities | X |  |  |  |  |
| Eligibility screen and recruitment of participants | X |  |  |  |  |
| Informed consent |  | X |  |  |  |
| Allocation |  |  | X |  |  |
| **INTERVENTIONS:** |  |  |  |  |  |
| *Film-based intervention* |  |  |  |  |  |
| *Treatment as usual* |  |  |  |  |  |
| **ASSESSMENTS:** |  |  |  |  |  |
| *Physical abuse, emotional abuse, positive parenting, attitude towards physical punishment, caregiver involvement in learning, caregiver psychological distress, caregiver coping, family functioning, social support, lifetime trauma exposure, daily stressors, demographic characteristics* |  | X |  |  |  |
| *Physical abuse, emotional abuse, positive parenting, attitude towards physical punishment, caregiver involvement in learning and education, parenting knowledge, caregiver psychological distress, caregiver coping, family functioning, social support, exposure and reactions to film intervention* |  |  |  | X |  |
| *Physical abuse, emotional abuse, positive parenting, attitude towards physical punishment, parenting knowledge, caregiver psychological distress, caregiver coping, family functioning, adverse and positive childhood experiences, child mental health* |  |  |  |  | X |
