# Supplementary material for: A film-based intervention to reduce child maltreatment among migrant and displaced families from Myanmar: Protocol of a pragmatic cluster randomized controlled trial
Source: PLoS One. 2023 Oct 30;18(10):e0293623. doi: 10.1371/journal.pone.0293623 (PMC10615270; doi:10.1371/journal.pone.0293623)
Supplement: S3 File — (PDF) [file pone.0293623.s003.pdf]

# Effectiveness of a film-based intervention to reduce child maltreatment among migrant and displaced families from Myanmar: study protocol for a cluster randomized controlled trial

## Background

Child maltreatment (defined as physical, sexual, and emotional abuse or neglect) is a global public health crisis with self-reported prevalence rates ranging from 22.6% for physical abuse and 36.3% for emotional abuse (Stoltenborgh et al., 2015). While child maltreatment is a global problem affecting both high-income and low- and middle-income countries (LMICs), research has shown that children living in LMICs – particularly those experiencing the intersections of poverty, armed conflict and forced displacement – may be at increased risk of exposure (Skeen & Tomlinson, 2013; Stark & Landis, 2016). The potentially lifelong and intergenerational impacts of child maltreatment and forced displacement are well-documented and include behavioral and emotional problems, poor school performance, substance abuse, and increased risk of future violence perpetration and victimization (Skeen & Tomlinson, 2013; Frounfelker et al., 2020).

Parenting interventions have been shown to reduce the risk of child maltreatment as well as positively impact a range of caregiver and child mental health and behavioral outcomes (Desai et al., 2017). However, the delivery of parenting support remains extremely limited in LMICs and particularly in humanitarian and displacement settings where the need is arguably the greatest (McCoy, Melendez-Torres, & Gardner, 2020; Gillespie et al., 2022). Scaled up delivery of parenting support to displaced families in LMICs is constrained by several factors including limited infrastructure and workforce, inadequate financial resources, and low levels of education and basic and digital literacy among the target population (Sanders et al., 2022).

The current study will evaluate the impact of a film-based intervention on reducing physical and emotional abuse among migrant and displaced families from Myanmar living in Tak province, Thailand. Film-based interventions can transcend literacy barriers and be scaled up to reach large audiences at relatively low cost with minimal infrastructure and human resource support (Botchway et al., 2017). However, there is a paucity of evidence regarding the effectiveness of film and other types of mass media to promote behavior change, particularly in LMICs. Notable exceptions include impact evaluations of the MTV show *Shuga* which found positive impacts on HIV prevention outcomes among young people in South Africa (Birdthistle et al., 2022), and a radio campaign in Burkina Faso which found an increase in antenatal help seeking behaviors (Sarrassat et al., 2018).

To our knowledge, this study will be the first randomized controlled trial evaluation of a film-based intervention to reduce child maltreatment among migrant and displaced families in a LMIC. This study is being implemented as part of the Global Parenting Initiative, a global consortium of academic and research institutions and implementing organizations aiming to provide access to free, evidence-based, playful parenting support to promote child learning and prevent violence at scale in the Global South.

## Methods

### Design

The study is a pragmatic, superiority cluster randomized controlled trial with 40 communities (clusters) randomized to the intervention or treatment as usual (TAU) in a 1:1 ratio. Families in communities allocated to the intervention arm will be invited to community film screening and post-screening discussion about positive parenting and coping strategies. Primary outcomes for the trial are (1) physical and emotional abuse; and (2) positive parenting behavior. Secondary outcomes include negative parenting behavior (e.g., hostility, indifference/neglect), caregiver attitude towards physical punishment, caregiver psychological distress, and family functioning. Exploratory outcomes include caregiver coping, behaviours to support early learning, social support, caregiver and adolescent health-related quality of life, and adolescent-reported mental health and psychosocial wellbeing. Outcomes will be assessed at baseline prior to randomization (T0), approximately 4 weeks post-intervention (T1), and 3-month follow up (T2), with the primary outcome point at T1. In addition to caregiver reports at all time points, we will assess a subsample of adolescents aged 12 to 18 years at T2 only. Qualitative group interviews with caregivers and adolescents will be conducted at T1 to examine their experiences of the intervention and perceived impacts on caregiver, child, and family outcomes. Participant recruitment, data collection, and intervention delivery are expected to begin in March 2023 and will continue on a rolling basis until complete (approximately December 2023).

### Aims and hypotheses

The primary study aim is to evaluate the effectiveness and cost-effectiveness of a film-based intervention to reduce physical and emotional abuse and increase positive parenting at approximately 4 weeks post-intervention. Secondary aims are to assess the impacts of the intervention on negative parenting behavior, caregiver attitude towards physical punishment, caregiver psychological distress, and family functioning from T0 to T1, and at T2. Additional aims are to explore intervention impacts on caregiver coping, behaviours to support early learning, social support, health-related quality of life, and adolescent mental health and psychosocial wellbeing, as well as potential treatment moderators (e.g., lifetime trauma exposure, length of displacement) and mediators (e.g., caregiver mental health).

Our primary hypothesis is that the intervention will be superior to TAU in reducing physical and emotional abuse and increasing positive parenting at 4 weeks post-intervention. Our secondary hypotheses are that the intervention will reduce negative parenting, caregiver acceptance of physical punishment, and caregiver psychological distress, as well as improve family functioning.

### Setting

The study will be conducted in Tak Province in Thailand, on the border with Myanmar. There is a long-standing history of migration from Myanmar into Tak Province due to decades of armed conflict and political and economic instability in Myanmar (Birk, Davison et al. 2021). As a result of the *coup d'état* in Myanmar in March 2021, the area has seen a significant influx of individuals from Myanmar across the border into Tak (UNHCR, 2022). A 2022 report indicates that Thailand hosts 658,023 individuals from populations of concern including an estimated 91,401 refugees from Myanmar who live in the 9 refugee camps along the border (UNHCR, 2022). However, this figure does not include the large number of migrants and displaced people from Myanmar who

live outside the camps. The 2019 Thailand Migration Report estimates that there are 3.9 million migrants living in Thailand, with approximately 70% originating from neighbouring Myanmar (United Nations Thematic Working Group on Migration in Thailand, 2019). At present there is no form of national legislation or legal framework regarding refugees and migrants. Many individuals from Myanmar lack work or residence permits in Thailand, resulting in risk for deportation, exploitative working conditions, and challenges in accessing education and other essential services (Tyrosvoutis, 2019; UNHCR, 2022). Reduced opportunities for mobility and employment as a result of the COVID-19 pandemic have further increased levels of poverty and food insecurity, which in turn has increased risk of child maltreatment and other negative child and family outcomes (International Organization for Migration, 2021).

Recent studies with migrant and displaced families from Myanmar in Tak Province highlight concerns about physical and emotional abuse in the home, child labor and neglect, as well as growing mental health difficulties such as depression and anxiety among both parents and children (Zar, Tyrosvoutis, & Costello, 2021; Birk et al., 2021). Results from the 2015 Myanmar Demographic Health Survey showed high prevalence of harsh discipline, with 74% of children aged 2 to 14 experiencing emotional abuse and 43% experiencing physical abuse (Ministry of Health and Sports & ICF, 2017). These findings suggest that the use of harsh punishment is widespread among the Myanmar population and may be further intensified by the chronic adversity experienced by migrant and displaced families living across the border in Thailand.

The study will be implemented in partnership with local non-governmental and community-based organizations Mae Tao Clinic, Help Without Frontiers Thailand Foundation, TeacherFOCUS, and Sermpanya Foundation, which all have extensive experience providing health, education, and psychosocial support to migrant and displaced families from Myanmar in Tak province.

## Intervention

*Film-based intervention*

The film-based intervention consists of a 60-minute live action narrative film developed in partnership with Sermpanya Foundation, a local non-governmental organization that works with refugees and migrants to develop, produce, and screen educational films on the Thailand-Myanmar border. The film portrays positive parenting skills, family relationships, and mental health coping strategies drawn from the Parenting for Lifelong Health (PLH) program (McCoy et al., 2021; Cluver et al., 2018). To inform content and ensure cultural and contextual relevance, we conducted formative qualitative research on parenting and mental health with caregivers and adolescents from Myanmar living in Tak province. Script development was led by a refugee from Myanmar and drafts of the script were reviewed by an advisory committee formed for this study and by a group of parents and caregivers from Myanmar currently living in Tak province. The characters in the film are played by families from Myanmar living in the communities depicted in the film.

Sermpanya Foundation will conduct mobile cinema screenings of the film in community locations that can accommodate large groups and that are accessible and safe for migrant and displaced families. Immediately after the film screening, trained community facilitators (e.g., local partner organization staff, community volunteers, teachers) will lead a structured discussion with audience members to reinforce the key messages and skills depicted in the film and to promote audience

discussion and engagement through open-ended questions and brief quizzes. The audience discussion will last approximately 30 minutes.

To maximize exposure in communities randomized to receive the intervention, we will develop promotional materials such as posters and flyers to be displayed in community spaces (e.g., schools, clinics, religious buildings) and distributed by local partners and community leaders. All adult and child residents of intervention group communities (not only those enrolled in the study) will be welcome to attend the film screening. To increase the likelihood that study participants attend the film screening, we will use phone calls, text messages, and home visits to send targeted invitations and reminders encouraging them to attend.

*Treatment as usual*

Treatment as usual will consist of information about existing services related to child protection and safeguarding and related education and health services. In collaboration with local partners, we will develop a list of services with brief descriptions including information on how to access services. Families in both the intervention and control groups will receive this information via handouts distributed at the time of the baseline assessment. Families in the intervention and control groups will not be prevented from participating in any other interventions or services during the course of the study.

**Community selection, randomization and blinding**

An initial list of potential communities in 4 districts in Tak province (Mae Sot, Pop Phra, Mae Ramat, Tha Song Yang) will be generated from a database of migrant learning centres (nonformal community-based schools attended by migrant and displaced children and youth from Myanmar) available from the Migrant Educational Coordination Center (Migrant Educational Coordination Center, 2022). Based on discussions with implementing partners, we will purposively select 40 communities that have at least 70 migrant and displaced families from Myanmar with children aged 4 to 18 years. Following community selection, we will conduct meetings with formal and informal community leaders and stakeholders (e.g., village leaders, teachers) to provide information about the study and gain their approval and support.

The 40 communities will be randomly allocated to intervention and control arms in a 1:1 ratio, stratified by district (urban Mae Sot vs. other districts). Stratification by district will minimize imbalance between groups due to factors that may be associated with the study outcomes (e.g., household income, caregiver education), thereby reducing between-cluster variability and increasing statistical power. Following Cochrane guidelines and to reduce the possibility of recruitment bias, randomization will be performed by the trial statisticians (QH, GJMT) after site selection and recruitment using a random number generator in Excel.

To facilitate implementation, data collection and intervention delivery will be conducted on a rolling basis to pairs of intervention and control group communities. Intervention and control group communities will be paired to ensure separation by a geographical buffer (e.g., at least one community/village or natural structure) to reduce the potential for spillover effects. Furthermore, as many families from Myanmar do not have legal status in Thailand, travel between communities tends to be limited. To reduce risk of bias related to time/seasonality, the order of rollout to pairs of intervention and control sites will be randomized.

The trial statisticians (QH, GJMT) conducting randomization and analyses will be blinded to condition. Blinding of participants and implementers will not be possible as they will know whether or not they are receiving the film-based intervention. However, data collection staff will be blinded at baseline data collection (T0) and every effort will be made to keep them blinded at T1 and T2, although it is plausible that data collection staff will hear from participants and implementers which communities received the intervention.

## Participants

*Inclusion/exclusion criteria*
Participants are caregivers of at least one child aged 4 to 18 years old and adolescents aged 12 to 18 years old. Caregivers are defined as parents or guardians with primary responsibility for the care of a minor child under the age of 18 years living in the same household. There is no requirement for a biological relationship between the caregiver and child. Caregivers will be recruited if they meet the following inclusion criteria: primary caregiver of at least one child aged 4 to 18 years; from Myanmar; currently residing in the study site; and, conversant in Burmese. In addition, adolescents aged between 12 and 18 years who reside in the same household as the caregiver will be eligible for assessments at T2. Individuals will be excluded if they have significant or severe cognitive, neurological, or developmental impairments that render them unable to provide informed consent, as assessed by data collection staff. Caregivers and adolescents who reside in institutions or group homes (e.g., boarding houses, orphanages) will be excluded.

*Recruitment*
Formal and informal community leaders and other community members who are knowledgeable about children and families (e.g., teachers, community volunteers working with implementing partners) will be asked to invite all caregivers who meet the inclusion criteria to attend a community meeting. At the community meeting, study staff will provide information about the study and invite interested caregivers to participate in a brief screening to confirm eligibility. Study staff will register the names and contact information of caregivers who meet the inclusion criteria to schedule assessments. Study staff will also call or send a message to registered caregivers the day before the scheduled assessment date to remind them to attend. In addition to the community meeting described above, targeted outreach to families will be conducted by teachers and community volunteers as needed to ensure the target sample size is achieved in each study site.

## Informed consent and data collection

We will pilot test the feasibility of using Computer Assisted Personal Interviewing (CAPI) or Audio Computer Assisted Self Interviewing (ACASI) to administer the surveys. If using CAPI, data collection staff will read out the study information sheet to participants and provide opportunities for participants to ask questions. Participants will then be asked to check the appropriate box on the informed consent statement on an electronic tablet to indicate their consent (if given). Data collection staff will then read out the survey items on the tablet and enter the participants' response on the tablet. The same informed consent procedure will be followed if ACASI is used; however, participants will be provided with a tablet and headphones so that they can read and listen to the audio recording of the survey items before entering their response on the tablet themselves. Data collection staff will begin with a demonstration of the tablet and ACASI,

followed by a brief practice session completed by participants. Staff will be available to answer any questions and provide assistance if required. Assessments will be conducted in a private place that is convenient and acceptable to participants (e.g., home, school). In the case that participants do not attend a scheduled assessment, three attempts via phone call and/or home visit will be made to contact them to schedule a new appointment.

Prior to the study, locally hired data collection staff will receive training on research ethics, informed consent procedures, interview techniques, managing participant distress, procedures for reporting adverse events, and data management and security. Ongoing training and supervision of data collection staff will be conducted by the locally based research coordinators (KZL, SEP).

**Adolescent sample**

Adolescent children of participating caregivers will be eligible for inclusion in assessments at T2 if they are aged between 12 and 18 years old. At the end of the endline survey (T1), caregivers will be asked if they consent for their adolescent child to participate in the study and to provide their child's name. Assent and data collection procedures for adolescents will be the same as those described above for caregivers.

**Incentives**

Caregiver and adolescent participants will receive a token of appreciation (e.g., mobile phone credit, stationery) with a value of 120 THB (approximately USD$3) at each assessment point. In addition, a lottery will be conducted in each community after each round of data collection for a chance to win one of 5 prizes worth a total of 1,000 THB (approximately USD $27).

**Outcome measures**

Existing Burmese translations of measures were obtained from study authors where available. All other measures were translated into Burmese following recommended procedures including forward translation to Burmese, back translation to English by an independent translator, and consultations with bilingual team members to review and resolve any discrepancies (Behr & Shishido, 2016). Survey instruments will be pilot tested with the target population to assess acceptability, relevance, comprehensibility, and length prior to data collection.

*Primary outcomes*

Physical and emotional abuse will be assessed using the International Society for Prevention of Child Abuse and Neglect Child Abuse Screening Tool (ICAST-Trial, 14 items) (Meinck et al., 2018). Positive parenting will be assessed using an adapted version of the Parent Behavior Inventory (PBI, 10 items) (Puffer et al., 2017). The PBI was previously developed and used by authors AS and EP for a trial of a family skills intervention on the Thailand-Myanmar border.

*Secondary outcomes*

Negative parenting will be assessed using items from the Hostility/Aggression, Indifference/Neglect, and Undifferentiated Rejection subscales of the Parental Acceptance and Rejection Questionnaire-Short Form (PARQ-SF, 16 items) (Khaleque & Rohner, 2012). The PARQ-SF was previously used by authors AS and EP for a trial of a family skills intervention on the Thailand-Myanmar border (Puffer et al., 2017). Internal consistency reliability for the combined scores from the negative subscales was 0.72 for caregiver report and 0.81 for child

report. Caregiver attitude towards physical punishment will be assessed by asking caregivers to indicate their agreement with a single item from the Myanmar Demographic Health Survey 2015 (*"Do you believe that in order to bring up your child properly, you need to physically punish him/her?"*) (Ministry of Health and Sports & ICF, 2017). Caregiver psychological distress will be assessed by the 10-item version of the Hopkins Symptom Checklist (HSCL) (Strand et al., 2003). The HSCL has been used extensively with refugees and migrants from Myanmar (Akiyama et al., 2013, Riley et al., 2020, Schweitzer et al., 2011). Family functioning will be assessed using 9 items from the Burmese Family Functioning Scale, developed by authors AS and EP from formative qualitative research and previously used in a trial of a family skills intervention on the Thailand-Myanmar border (Puffer et al., 2017).

*Exploratory outcomes*

Caregiver coping and stress management will be assessed by 5 items drawn from the Parent Behavior Inventory (e.g. *"In the past 4 weeks until now, I felt too busy, tired or stressed to spend time with my child."*) (Puffer et al., 2017). Caregiver behaviours to support early learning will be assessed by 6 items drawn from the Myanmar Demographic Health Survey 2015 (Ministry of Health and Sports & ICF, 2017) (e.g. *"In the past 7 days, how often did you read books to or look at picture books with your child?"*) and administered only to caregivers of children aged 4 to 8 years. To assess caregiver social support, we will use an abbreviated version of the Medical Outcomes Study Social Support Scale (MOS-SSS) consisting of 5 items from the tangible support, emotional-informational support, affectionate support, and positive social interaction support subscales (Gjesfjeld, Greeno, & Kim, 2008). The MOS-SSS was previously used in a study with Burmese adolescents living on the Thailand-Myanmar border (Akiyama et al., 2013). Caregiver and adolescent health-related quality of life will be assessed by the EQ-5D (Feng et al., 2021) and CHU9D (Stevens, 2012) respectively. Adolescent-reported mental health will be assessed by the Hopkins Symptom Checklist-37 (HSCL-37A), a modified version of the HSCL-25 for adolescents (Bean et al., 2007). The HSCL-37A assesses internalizing and externalizing symptoms and has been previously used with Burmese adolescents on the Thailand-Myanmar border (Akiyama et al., 2013). Adolescent-reported psychosocial wellbeing will be assessed by the Short Warwick Edinburgh Mental Wellbeing Scale (SWEMBS, 7 items) (Ng Fat et al., 2017). The Warwick Edinburgh Mental Wellbeing Scale has been used with adolescents in other conflict and displacement settings (Wu et al., 2018).

*Other measures*

Sociodemographic characteristics (e.g. age, education level, length of stay in Thailand), caregivers' adverse and benevolent childhood experiences, lifetime trauma exposure, and daily stressors will also be assessed to characterize the study sample and to examine potential moderators of treatment effects. Caregivers' adverse childhood experiences will be assessed using adapted items from the Adverse Childhood Experiences International Questionnaire (ACE-IQ) developed by the World Health Organization (WHO, 2018). Caregivers' benevolent childhood experiences will be assessed using adapted items from the Benevolent Child Experiences Scale, which assesses positive early life experiences in adults with histories of adversity. Caregivers' lifetime trauma exposure will be assessed using a traumatic events checklist adapted for this study using items from the Building a New Life in Australia study (Narayan et al., 2019) and the Harvard Trauma Questionnaire (Mollica, McDonald, Massagli, & Silove, 2004) and consulting with local partners. Each item is scored as "yes" or "no" for occurrence regardless of when or where it

occurred. Caregivers' experience of daily stressors will be assessed using items adapted from the Humanitarian Emergency Settings Perceived Needs Scale (HESPER) developed by the World Health Organization (WHO, 2011) with the addition of contextually relevant items developed from formative qualitative research. Adolescents' experience of stressful life events will be assessed using the Stressful Life Events Checklist (Bean et al., 2007), which was previously used in a study with Burmese adolescents living on the Thailand-Myanmar border (Akiyama et al., 2013).

Finally, T1 and T2 surveys will include assessment of exposure to the intervention (including in the control arm to assess spillover), recognition and retention of key intervention messages, identification with characters in the film, and whether participants attended the film screening with other family members or discussed the film with other family and community members.

**Sample size**

The sample size calculation was performed by QH and GJMT and based on a two-group comparison of one of the primary outcomes (physical and emotional abuse) assessed at T1. Assuming a significance level of 5% with an intra-cluster correlation coefficient (ICC) of 0.02 and a two-tailed test with 0.9 power, 40 clusters with 50 families per cluster would be required to detect an effect size of 0.2. The total sample size was set at 2,200 families (40 clusters with 55 families per cluster) to account for up to 10% attrition. Our estimate of expected effect size was based on a review of parenting interventions for child maltreatment prevention which found an average effect of 0.2 (Chen & Chan, 2016).

**Statistical analysis**

Trial results will be reported following the updated recommendations of the Consolidated Standards of Reporting Trials (CONSORT) 2010 statement: extension to cluster randomised trials. Initial analyses will compare baseline characteristics of participants across the two study arms as well as participants who completed T1 assessments and those who did not.

*Caregiver analyses.* Outcomes will be analysed using three-level models with measurement wave within participant within cluster. Level 1 will include a term for categorical time (T1 and T2); level 2 will include terms for caregiver and child age and gender, centred at the sample mean; and level 3 will include terms for intervention, stratification, and the interactions between intervention and categorical time. Thus, the test of intervention effectiveness is the interaction between intervention and categorical time. Crude (i.e. models including intervention, time, intervention by time interactions, and stratification) and adjusted (i.e. models additionally including level 2 covariates) mean differences in the primary outcomes between the two study arms at T1 will be derived from the mixed models using 95% CIs.

*Adolescent analyses.* Outcomes will be analysed using two-level models with adolescents nested within cluster. Because there are no baseline measures for this subgroup to test change over time, models for T1 and T2 outcomes will be estimated simultaneously in a multivariate framework. Level 1 will include terms for child age and gender, centred at the sample mean and assumed to be equal for both T1 and T2 outcomes; and level 2 will include terms for intervention and stratification. The term for stratification will be assumed to be equal for both T1 and T2 outcomes, whereas the intervention term will be estimated freely for both T1 and T2 random intercepts.

Primary analyses will be conducted using the intention to treat principle. To account for dropouts in the intention-to-treat analysis, the baseline measurement will be part of the repeated outcomes and estimation of the intervention effects will be via maximum likelihood. The impact of the missing data on the estimated intervention effect will be assessed by imputing missing outcome data (at least 10 imputations, using an unrestricted multilevel model with fully conditional specifications) using complete baseline and follow-up data and running the same models. Per-protocol analyses will also be conducted using only the data on participants who received the intervention as planned. All additional analyses, including analysis of potential moderators and mediators, will be detailed in a statistical analysis prior to unblinding. Across all analyses, two-tailed tests will be reported with a significance level of $p<0.05$.

*Cost effectiveness analysis*

Costs will be divided into set-up costs (e.g. initial script development and film production costs) and intervention delivery costs (e.g. travel, staffing, film screening equipment and supplies). Cost data will be collected from project budget and expenditure reports and verified through consultation with project staff. Cost effectiveness analysis will include the following phases: (1) review of budget/expenditure sheets and consultation with project staff; (2) calculating costs; (3) calculating effectiveness; and (4) calculating cost-effectiveness ratios to provide the cost per standard deviation unit change in primary outcomes.

**Process and qualitative evaluation**

Total attendance at community film screenings will be tracked by research staff conducting a headcount of all attendees midway through the film to provide an estimate of the number of adults and children present. To track attendance by study participants specifically, each adult entering the film screening will be asked if they participated in the baseline survey and if so, their names will be checked off a list. We will also ask whether study participants attended the film screening at the post-intervention assessment (T1). We will not collect identifying information from film screening attendees who are not enrolled in the study.

To assess fidelity of the post-film audience discussion, trained research staff will observe a random sample of 25% of community film screenings and complete a structured observation form to score which components of the discussion were completed and to what quality.

In addition, up to 15 focus group discussions will be conducted with a random sample of caregivers and adolescents who reported attending the film screenings to qualitatively examine their experience and opinions of the film, their perception of key messages or themes, and perceived impacts on their and their children's behavior and wellbeing. Focus group discussions will take place approximately 4 weeks (T1) post-intervention. Qualitative data will be analyzed using thematic content analysis (Braun & Clarke).

**Data management**

Assessments will be administered using Open Data Kit (ODK) on password-protected tablets and uploaded daily onto a secure encrypted server located at the University of Oxford. All submissions will be cross-checked before data are permanently removed from the tablets each day. Only authorized members of the research team will have access to data on the server. Data on the ODK server is automatically backed up daily, an additional weekly back up will be made in University

of Oxford OneDrive for business onto a password-protected OneDrive folder accessible only to authorized research team members. Any hard copies of forms will be scanned and uploaded to the server, after which they will be permanently destroyed. Transcriptions and audio recordings will also be stored in OneDrive, once audios have been verified, they will be deleted from recording devices.

All data will be de-identified prior to analysis. Participant responses will be identified only by a unique study code. Information linking the unique study code to participants' identifying information such as names and contact information will be stored separately on a password-protected OneDrive folder accessible only to authorized research team members and destroyed at the conclusion of the study. Transcripts of audio recordings of focus group discussions will be anonymized by removing all personal identifying data. Audio recordings will be destroyed upon verification of the transcripts. De-identified data will be stored securely in perpetuity for research purposes only. Participants will be able to notify the study team if they want to withdraw their data until the personal linked data is destroyed.

**Trial and adverse events monitoring**

A trial management committee consisting of principal investigators, co-investigators, and research coordinators will monitor the implementation of study procedures. Any deviations from the study protocol will be reported to the trial management committee and to the research ethics committees as necessary. Any modifications to the protocol will be submitted to the research ethics committees for approval and trial protocols will be updated online on the relevant registries.

The research team will be trained on a safety protocol developed for this study that will include information on reporting and referral procedures if a safety concern is identified. Local partner organizations also have a Child Safeguarding Policy that includes a protocol for reporting suspected child maltreatment, exploitation, or any other violation of the policy. All staff will be required to adhere to this policy. Data collection staff will be trained to record and report all adverse events (e.g. extreme participant distress, child safeguarding concerns) to the local research coordinators (KZL, SEP) within 24 hours. A committee comprised of the PIs (AS, TJ), local co-investigator and protection lead (NNO), and research coordinators will be responsible for reviewing all reported adverse events within 48 hours and determining any necessary action, which may include referral to the local Child Protection Unit. Information is included in the informed consent form to notify participants that disclosures of harm to self or others may be reported.

To reduce risk of participant distress, data collection staff will be trained to remind participants that they can choose to skip any questions or withdraw from the study at any time with no negative consequences. All participants will be provided information about available mental health and psychosocial support services during each assessment point. No interim analyses are planned. The local research coordinators are responsible for ensuring timely follow up of any adverse events.

**COVID-19 protocols**

Study procedures will follow all COVID-19 public health regulations in Thailand at the time of data collection. To reduce the risk of COVID-19 transmission, masks and hand sanitizer will be distributed to all research participants and staff, and gatherings (e.g. community meetings) will be conducted in large, well-ventilated areas and in accordance with local public health regulations.

Participants and staff will be asked screening questions and anyone reporting symptoms or close contacts will be referred to health services or asked to isolate depending on local guidelines.

**Dissemination**

Results of this study will be communicated through a variety of channels to reach multiple audiences. To share results with participants and community members, we will create brief reports and presentations designed for non-specialists that present the main findings without the use of technical language or jargon. For academic audiences, results will be published through peer-reviewed journal articles and presented at academic conferences. For practitioners and policy makers within Thailand, the region, and globally, results will be disseminated via research reports, policy briefs, infographics, webinars, and targeted meetings and conferences.

**Discussion**

This study is the first known randomized controlled trial of a universal film-based intervention to prevent child maltreatment in a forced displacement setting. The aim is to provide a light-touch, low-cost, and easily scalable intervention that can be delivered at a population-level in low resource and low literacy settings. Using film as an intervention modality may address barriers to equitable access to parenting and psychosocial support in LMICs. If effectiveness is demonstrated, there is potential for the intervention to be adapted and scaled out to families and communities experiencing adversity in other contexts.

## References

Akiyama, T., et al. (2013). "Mental health status among Burmese adolescent students living in boarding houses in Thailand: a cross-sectional study". *BMC Public Health*, 13(1): 1-12.

Bean, T., Derluyn, I., Eurelings-Bontekoe, E., Broekaert, E., & Spinhoven, P. (2007). Validation of the multiple language versions of the Hopkins Symptom Checklist-37 for refugee adolescents. *Adolescence*, *42*(165), 51.

Behr, D., & Shishido, K. (2016). The translation of measurement instruments for cross-cultural surveys. *The SAGE handbook of survey methodology*, 269-287.

Birdthistle, I., Mulwa, S., Sarrassat, S., Baker, V., Khanyile, D., O'Donnell, D., ... & Cousens, S. (2022). Effects of a multimedia campaign on HIV self-testing and PrEP outcomes among young people in South Africa: a mixed-methods impact evaluation of 'MTV Shuga Down South'. *BMJ global health*, 7(4), e007641.

Birk, S. K., et al. (2021). "Perceptions of child physical discipline among Burmese migrants living in Mae Sot, Thailand." Social Sciences & Humanities Open 4(1): 100234.

Botchway, S., Bettiol, S. S., van Schalkwyk, M., Flodgren, G., & Hoang, U. (2017). Films for public health: developing the evidence base for films to support public health goals. Perspectives in Public Health, 137(5), 260-261.

Braun, V., & Clarke, V. (2006). Using thematic analysis in psychology. *Qualitative research in psychology*, *3*(2), 77-101.

Chen M, Chan KL. Effects of parenting programs on child maltreatment prevention: a meta-analysis. *Trauma Violence Abuse*. 2016;17(1):88–104.

Cluver, L. D., Meinck, F., Steinert, J. I., Shenderovich, Y., Doubt, J., Romero, R. H., ... & Gardner, F. (2018). Parenting for lifelong health: a pragmatic cluster randomised controlled trial of a non-commercialised parenting programme for adolescents and their families in South Africa. *BMJ global health*, *3*(1), e000539.

Coore Desai, C., Reece, J. A., & Shakespeare-Pellington, S. (2017). The prevention of violence in childhood through parenting programmes: a global review. *Psychology, Health & Medicine*, *22*(sup1), 166-186.

Feng, Y. S., Kohlmann, T., Janssen, M. F., & Buchholz, I. (2021). Psychometric properties of the EQ-5D-5L: a systematic review of the literature. *Quality of Life Research*, *30*(3), 647 673.

Frounfelker, R. L., Miconi, D., Farrar, J., Brooks, M. A., Rousseau, C., & Betancourt, T. S. (2020). Mental health of refugee children and youth: Epidemiology, interventions, and future directions. *Annual Review of Public Health*, *41*, 159-176.

Gillespie, S., Banegas, J., Maxwell, J. *et al.* Parenting Interventions for Refugees and Forcibly Displaced Families: A Systematic Review. *Clin Child Fam Psychol Rev* **25**, 395–412 (2022). https://doi.org/10.1007/s10567-021-00375-z

International Organization for Migration (2022). Myanmar Crisis Response Plan 2022 – Myanmar and Thailand https://crisisresponse.iom.int/sites/g/files/tmzbdl1481/files/appeal/pdf/2022_Myanmar_Crisis_Response_Plan_2022__Myanmar_and_Thailand.pdf.

Gjesfjeld, C. D., et al. (2008). "A confirmatory factor analysis of an abbreviated social support instrument: The MOS-SSS." Research on Social Work Practice 18(3): 231-237.

Khaleque, A., & Rohner, R. P. (2012). Pancultural associations between perceived parental acceptance and psychological adjustment of children and adults: A meta-analytic review of worldwide research. *Journal of cross-cultural Psychology*, *43*(5), 784-800.

McCoy, A., Melendez-Torres, G. J., & Gardner, F. (2020). Parenting interventions to prevent violence against children in low-and middle-income countries in East and Southeast Asia: a systematic review and multi-level meta-analysis. *Child abuse & neglect*, *103*, 104444.

McCoy, A., Lachman, J. M., Ward, C. L., Tapanya, S., Poomchaichote, T., Kelly, J., ... & Gardner, F. (2021). Feasibility pilot of an adapted parenting program embedded within the Thai public health system. *BMC public health*, *21*(1), 1-19.

Meinck, F., Boyes, M. E., Cluver, L., Ward, C. L., Schmidt, P., DeStone, S., & Dunne, M. P. (2018). Adaptation and psychometric properties of the ISPCAN Child Abuse Screening Tool for use in trials (ICAST-Trial) among South African adolescents and their primary caregivers. *Child abuse & neglect*, *82*, 45-58.

Migrant Educational Coordination Center (2022). Migrant Learning Centres Along Tak Province. https://datastudio.google.com/u/0/reporting/7759919f-aca9-4483-b36a 8540e5feee01/page/NsJuC.

Ministry of Health and Sports & ICF. (2017). Myanmar Demographic and Health Survey 2015 16. Nay Pyi Taw, Myanmar, MoHS and ICF.

Mollica R, McDonald L, Massagli M, Silove D. *Measuring trauma, measuring torture.* Cambridge, Mass: Harvard Program in Refugee Trauma, 2004.

Narayan, A. J., Rivera, L. M., Bernstein, R. E., Harris, W. W., & Lieberman, A. F. (2018). Positive childhood experiences predict less psychopathology and stress in pregnant women with childhood adversity: A pilot study of the benevolent childhood experiences (BCEs) scale. Child abuse & neglect, 78, 19-30.

Ng Fat, L., Scholes, S., Boniface, S., Mindell, J., & Stewart-Brown, S. (2017). Evaluating and establishing national norms for mental wellbeing using the short Warwick–Edinburgh Mental Well-being Scale (SWEMWBS): findings from the Health Survey for England. *Quality of Life Research*, *26*(5), 1129-1144.

Puffer ES, Annan J, Sim AL, Salhi C, Betancourt TS (2017) The impact of a family skills training intervention among Burmese migrant families in Thailand: A randomized controlled trial. PLoS ONE 12(3): e0172611.

Riley, A., Varner, A., Ventevogel, P., Taimur Hasan, M. M., & Welton-Mitchell, C. (2017). Daily stressors, trauma exposure, and mental health among stateless Rohingya refugees in Bangladesh. *Transcultural Psychiatry*, *54*(3), 304–331. https://doi.org/10.1177/1363461517705571

Sanders, M.R., Divan, G., Singhal, M. *et al.* Scaling Up Parenting Interventions is Critical for Attaining the Sustainable Development Goals. *Child Psychiatry Hum Dev* **53**, 941–952 (2022). https://doi.org/10.1007/s10578-021-01171-0

Sarrassat, S., Meda, N., Badolo, H., Ouedraogo, M., Some, H., Bambara, R., ... & Head, R. (2018). Effect of a mass radio campaign on family behaviours and child survival in Burkina Faso: a repeated cross-sectional, cluster-randomised trial. The Lancet Global Health, 6(3), e330-e341.

Schweitzer, R. D., Brough, M., Vromans, L., & Asic-Kobe, M. (2011). Mental health of newly arrived Burmese refugees in Australia: Contributions of pre-migration and post-migration experience. *Australian & New Zealand Journal of Psychiatry*, *45*(4), 299–307. https://doi.org/10.3109/00048674.2010.543412

Stevens, K. (2012). Valuation of the child health utility 9D index. *Pharmacoeconomics*, *30*(8), 729-747.

Stoltenborgh, M., Bakermans-Kranenburg, M. J., Alink, L. R., & van IJzendoorn, M. H. (2015). The prevalence of child maltreatment across the globe: Review of a series of meta-analyses. *Child Abuse Review, 24*(1), 37-50.

Strand, B. H., Dalgard, O. S., Tambs, K., & Rognerud, M. (2003). Measuring the mental health status of the Norwegian population: a comparison of the instruments SCL-25, SCL-10, SCL-5 and MHI-5 (SF-36). *Nordic journal of psychiatry*, *57*(2), 113-118.

Sim, A., Annan, J., Puffer, E., Salhi, C., & Betancourt, T. (2014). Building happy families: Impact evaluation of a parenting and family skills intervention for migrant and displaced Burmese families in Thailand. *New York: International Rescue Committee*

Stark, L., & Landis, D. (2016). Violence against children in humanitarian settings: A literature review of population-based approaches. *Social Science & Medicine*, *152*, 125-137.

Stoltenborgh, M., Bakermans-Kranenburg, M. J., Alink, L. R., & van IJzendoorn, M. H. (2015). The prevalence of child maltreatment across the globe: Review of a series of meta analyses. *Child Abuse Review*, *24*(1), 37-50.

Tyrosvoutis, G. (2019). Bridges: Participatory action research on the future of migrant education in Thailand. Help without Frontiers. Retrieved from https://helpwithout frontiers.org/sites/helpwithoutfrontiers.org/files/resources-docs/eng_full_report_bridges.pdf.

United Nations High Commissioner for Refugees (2022). Refugee Influx: Tak province, Thailand - Inter-Agency Operational Update. Retrieved from https://data.unhcr.org/en/documents/details/90357

United Nations Thematic Working Group on Migration in Thailand (2019). Thailand Migration Report 2019. https://thailand.un.org/sites/default/files/2020-06/Thailand-Migration Report-2019.pdf.

World Health Organization & King's College London (2011). The Humanitarian Emergency Settings Perceived Needs Scale (HESPER): Manual with Scale. Geneva: World Health Organization

World Health Organization. (2018). Adverse childhood experiences international questionnaire. *Adverse childhood experiences international questionnaire (ACE-IQ)*, 245-258.

Wu, Q., Ge, T., Emond, A., Foster, K., Gatt, J. M., Hadfield, K., ... & Wouldes, T. A. (2018). Acculturation, resilience, and the mental health of migrant youth: a cross-country comparative study. *Public health*, *162*, 63-70.

Zar, T., Tyrosvoutis, G., & Castello, I. (2021). Improving protection for migrant children during the COVID-19 pandemic. Mae Sot, Thailand, Help Without Frontiers Thailand Foundation and TeacherFOCUS.
